# Impact of total intravenous anesthesia and total inhalation anesthesia as the anesthesia maintenance approaches on blood glucose level and postoperative complications in patients with type 2 diabetes mellitus: a double-blind, randomized controlled trial

**DOI:** 10.1186/s12871-023-02199-6

**Published:** 2023-08-09

**Authors:** Xinghui Xiong, Yong He, Cheng Zhou, Qin Zheng, Chan Chen, Peng Liang

**Affiliations:** 1 Department of Anesthesiology, West China Hospital, Sichuan University, Chengdu, 610041 Sichuan China; 2 Department of Laboratory Medicine, West China Hospital, Sichuan University, Chengdu, 610041 Sichuan China; 3 Laboratory of Anesthesia and Critical Care Medicine, West China Hospital, National-Local Joint Engineering Research Centre of Translational Medicine of Anesthesiology, Sichuan University, Chengdu, 610041 Sichuan China; 4 Day Surgery Center, West China Hospital, Sichuan University, Chengdu, 610041 Sichuan China

**Keywords:** Total intravenous anesthesia, Total inhalation anesthesia, Blood glucose level, Diabetes mellitus, Complication

## Abstract

**Background:**

Diabetes mellitus is a prevalent metabolic disease in the world. Previous studies have shown that anesthetics can affect perioperative blood glucose levels which related to adverse clinical outcomes. Few studies have explored the choice of general anesthetic protocol on perioperative glucose metabolism in diabetes patients. We aimed to compare total intravenous anesthesia (TIVA) with total inhalation anesthesia (TIHA) on blood glucose level and complications in type 2 diabetic patients undergoing general surgery.

**Methods:**

In this double-blind controlled trial, 116 type 2 diabetic patients scheduled for general surgery were randomly assigned to either the TIVA group or TIHA group (n = 56 and n = 60, respectively). The blood glucose level at different time points were measured and analyzed by the repeated-measures analysis of variance. The serum insulin and cortisol levels were measured and analyzed with t-test. The incidence of complications was followed up and analyzed with chi-square test or Fisher’s exact test as appropriate. The risk factors for complications were analyzed using the logistic stepwise regression.

**Results:**

The blood glucose levels were higher in TIHA group than that in TIVA group at the time points of extubation, 1 and 2 h after the operation, 1 and 2 days after the operation, and were significantly higher at 1 day after the operation (10.4 ± 2.8 vs. 8.1 ± 2.1 mmol/L; *P* < 0.01). The postoperative insulin level was higher in TIVA group than that in TIHA group (8.9 ± 2.9 vs. 7.6 ± 2.4 IU/mL; *P* = 0.011). The postoperative cortisol level was higher in TIHA group than that in TIVA group (15.3 ± 4.8 vs. 12.2 ± 8.9 ug/dL ; *P* = 0.031). No significant difference regarding the incidence of complications between the two groups was found based on the current samples. Blood glucose level on postoperative day 1 was a risk factor for postoperative complications (OR: 1.779, 95%CI: 1.009 ~ 3.138).

**Conclusions:**

TIVA has less impact on perioperative blood glucose level and a better inhibition of cortisol release in type 2 diabetic patients compared to TIHA. A future large trial may be conducted to find the difference of complications between the two groups.

**Trial registration:**

The protocol registered on the Chinese Clinical Trials Registry on 20/01/2020 (ChiCTR2000029247).

## Introduction

It was reported that 8.3% of the population in the United States is diabetic, while in China, the estimated prevalence of diabetes in adults is 10.9% [1, 2]. More and more diabetes patients need to be treated with surgery in the rest of their lives, especially many abdominal surgeries such as liver surgery, gastrointestinal surgery, and biliary surgery [3, 4]. Perioperative hyperglycemia is one of the consequences resulted from the metabolic and endocrine dysregulation in response to surgical stress [5]. Studies have shown that poor perioperative glycemic control in diabetic patients may lead to adverse clinical outcomes or even worse, significantly shorter life expectancy [6–10]. Patients with diabetes or preoperative hyperglycemia have a higher rate of perioperative mortality than the general population, and the perioperative mortality rate is five times higher in diabetic patients than in non-diabetic patients [2, 7–11]. In addition, hemoglobinA_1c_ (HbA_1c_) reflects the blood glucose control recently in diabetics, and is associated with complications and readmission rates [12, 13].

The effect of anesthetics on the intraoperative blood glucose level and the degree of the surgical stress caused by anesthetic agents plays a crucial role in the postoperative recovery of the patients. Several studies from animal and human have revealed that volatile anesthetics could impair glucose tolerance and suppress insulin secretion, which ultimately resulted in perioperative hyperglycemia [14–19]. In contrast, it has been reported that high-dose propofol infusion decreased glycemic and norepinephrine levels, suppressed intraoperative stress, and reduced the incidence of intraoperative disorders of glucose metabolism in diabetic patients [20, 21]. Volatile anesthetics were more likely to cause perioperative hyperglycemia, cortisol increase, and insulin reduction than propofol in non-diabetic patients [22].

A recent retrospective study showed that sevoflurane and propofol were comparable in terms of incidence and clinical outcomes with respect to perioperative hyperglycemia in diabetes patients undergoing pulmonary surgery [23]. It is known to all that total intravenous anesthesia (TIVA) and total inhalation anesthesia (TIHA) are two common general anesthetic protocols. To our knowledge, there is no randomized controlled trial on the selection of general anesthetic protocol (TIVA vs. TIHA) on perioperative glucose metabolism in diabetes patients undergoing general surgery. We hypothesized that those diabetes patients receiving TIVA might have lower perioperative blood glucose level and better outcome. Therefore, we conducted a randomized controlled study focused on comparison of the effects of TIVA (propofol) and TIHA (desflurane) on perioperative glucose, cortisol, insulin levels, and postoperative complications in diabetic patients undergoing general surgery.

## Methods

### Trial design

This randomized controlled single-center, double-blind, parallel trial was approved by the Biomedical Ethics Committee of West China Hospital of Sichuan University on 18/12/2019 (2019 − 928), and written informed consent was obtained from all subjects participating in the trial. The trial was registered prior to patient enrollment on the Chinese Clinical Trials Registry on 20/01/2020 (ChiCTR2000029247). Our study was conducted based on a prespecified protocol from January to October 2020 in accordance with the Helsinki Declaration-2013 [24]. Sex and/or gender were accounted for in the design of the study, the proportion of female patients was consistent in both groups.

### Participants

Potentially eligible patients were screened according to the inclusion and exclusion criteria. All adult type 2 diabetes patients (18–90 year old) with class I, II, or III based on the American Society of Anesthesiologists (ASA) physical status undergoing elective general surgery (≥ 2 h) were screened for inclusion according to the study protocol. Exclusion criteria included severe systemic diseases, metabolic disorders, diabetic ketoacidosis or hyperglycemia, diabetic neuropathy, hepatic and/or renal dysfunction, neuromuscular disease, and pancreatic cancer or islet cell tumor. All participants had to understand, express, and write Chinese and give written informed consent.

### Randomisation and blinding

The patients were randomized at 1:1 ratio to receive total intravenous anesthesia (TIVA group) or total inhalation anesthesia (TIHA group) by using the SPSS software. The center for Evidence-Based Medicine provided sequentially numbered opaque sealed envelopes to achieve allocation concealment. These sealed envelopes were kept by the screener who was not implementation of the interventions. The envelopes corresponding to the patient’s serial number were sent to the anesthesiologist after recruitment. Then the anesthesiologist opened the envelope and administered the appropriate anesthesia protocol (TIVA or TIHA) according to the card.

The randomization allocation schedule was blinded to the outcome evaluators who were not involved in the implementation of the interventions. Participants were blinded to the allocation and did not know the anesthetic protocol they received. It was impossible to blind anesthesiologists from the interventional schemes. However, anesthesiologists were not involved in the measurement or statistical analysis of outcomes and were required not to reveal the details of the anesthetic protocol to outcome evaluators or participants. The statistician was blinded to the group allocation until completion of the statistical analyses. The randomization allocation schedule was also blinded to the outcome evaluators, participants and statisticians until completion of the study.

### Intervention and measurement

Oral antidiabetic medications were continued in diabetes patients on the day before surgery, and were discontinued on the day of surgery. The doses of long-acting insulin administered should be reduced by 50–75% on the night before surgery to avoid hypoglycemia during a prolonged fast. Half doses of basal insulin were administered the morning of surgery [13, 25].

The patient was catheterized on radial artery under local anesthesia before induction, and blood was collected to measure the baseline data of relevant outcomes (blood glucose, insulin, and cortisol levels). Midazolam 0.04 mg/kg, sufentanil 0.4 µg/kg, propofol 2 mg/kg and cis-atracurium 0.2 mg/kg were administered for induction of conventional general anesthesia. Endotracheal intubation was performed after mask-assisted ventilation for 3 min.

For patients in the TIHA group, anesthesia was maintained with inhaled desflurane (1.0 to 1.5 minimum alveolar concentrations) and intravenous remifentanil infusion (0.12 to 0.24 µg·kg^–1^·min^–1^). For patients in the TIVA group, anesthesia was maintained by total intravenous anesthesia with propofol infusion (4 to 12 mg·kg^–1^·h^–1^) and remifentanil infusion (0.12 to 0.24 µg·kg^–1^·min^–1^). No limitations for the use of muscle relaxant and vasoactive medications were applied. However, glucose-containing fluids, glucocorticoid drugs and nonsteroidal analgesics were not allowed during the surgery. The anesthesiologist adjusted the drug dose according to the intensity of the surgical stimulus, patient’s vital signs, and the value of EEG bispectral index (BIS, maintained within 40 ~ 60).

Intraoperatively monitored parameters included heart rate, invasive blood pressure, pulse oxygen saturation (S_P_O2), BIS, and end-tidal gas. All patients were ventilated in volume-controlled mode with protective lung ventilation strategy (tidal volume: 6-8ml/kg; PEEP: 4–8 cmH_2_O; FiO_2_: 50%) [26]. The heart rate and blood pressure were continuously monitored and recorded at each time point during the operation, and maintained within the range of ± 20%. Vasoactive drugs were used if necessary.

Patients controlled analgesia, with a sufentanil infusion pump was used in the first 72 h after surgery. Dezocine were used for pain relief 3 days after the surgery. Postoperative use of hormones, non-steroidal anti-inflammatory drugs were avoided.

If the intraoperative glucose level was much higher than 10 mmol/L, an advisable dose of insulin should be given without causing hypoglycemia [13]. If the intraoperative blood glucose level was below 4 mmol/L, a 10% glucose solution was administrated. The blood glucose was measured again 30 min after treatment and adjusted accordingly with further insulin or 10% glucose solution.

## Outcomes

The primary outcomes are the blood glucose levels at different time points: preoperative(T0), immediate intubation (T1), skin incision (T2), 1, 2 and 3 h after the start of the operation (T3, T4 and T5, respectively), suturing (T6), extubation (T7), 1 h after the operation (T8) ,2 h after the operation (T9), 1 and 2 days after the operation (T10, T11). ​Secondary outcome measures included serum insulin and cortisol levels 30 min before and 30 min after surgery, and the incidence of postoperative complications. The complications were assessed by bedside or over telephone on the 1st, 3rd, 7th, and 30th post-operative day, including myocardial infarction (MI), stroke, acute kidney injury, renal failure, anastomotic fistula, stress ulcer, incision infection, arrhythmia, hypoglycemia, hyperglycemia, postoperative pulmonary complications (PPCS), and death. These postoperative complications were related to the functions of vital organs such as heart, lung, brain and kidney, and were positively associated with intense stress response during surgery. Postoperative complications were assessed according to the criteria from the European Perioperative Clinical Outcome (EPCO) and PPCS definitions [27, 28].

### Sample size

The sample size was corrected for repeated measures by blood glucose level at 11 different time points based on the results of the pilot trial with SPSS software. According to the pilot trial, the blood glucose level on the postoperative day 1 was largely adequate to prove a difference between the two groups, and the mean [SD] blood glucose levels were (TIVA 10.9 ± 2.2 vs. TIHA 11.7 ± 2.4 mmol/L ). The method used for sample size determination was matched-pair design formula with a type I error of 5% (α = 0.05) and 90% power (β = 0.1). The result showed that at least 51 patients were required in each group to show the differences of blood glucose level between the groups. Considering a 10% loss-to-follow-up rate, the final sample size was determined to be minimally 56 patients each group.

### Statistical analysis

The Statistical analysis of our study was achieved based on a prespecified analysis plan [24]. All data were analyzed with IBM SPSS Statistics 26.0 (IBM Corp., New York, NY, USA). Shapiro-Wilk test was used to test the normality of the data. For continuous variables, mean [SD] and 95% confidence interval (CI), or median (interquartile range) were used to represent the data, and were analyzed by T-test or Mann-Whiney U test. The blood glucose levels at different time points were analyzed by the repeated-measures analysis of variance (ANOVA). Time point and group allocation were the two factors analyzed during the intergroup comparisons. Hot-deck imputation strategy was used to treat patients with missing data. The serum insulin and cortisol levels were analyzed with t-test. The incidence of complications was presented as frequency (%), and analyzed with chi-square test or two-tailed Fisher’s exact test as appropriate. The risk factors for complications were analyzed using the logistic stepwise regression. A univariate regression was performed, and then those variables with *P*-value < 0.1 were included in multivariate logistic regression. The Bonferroni test was used in post hoc analysis to detect at which moment the difference was.

## Results

**Fig. 1:**
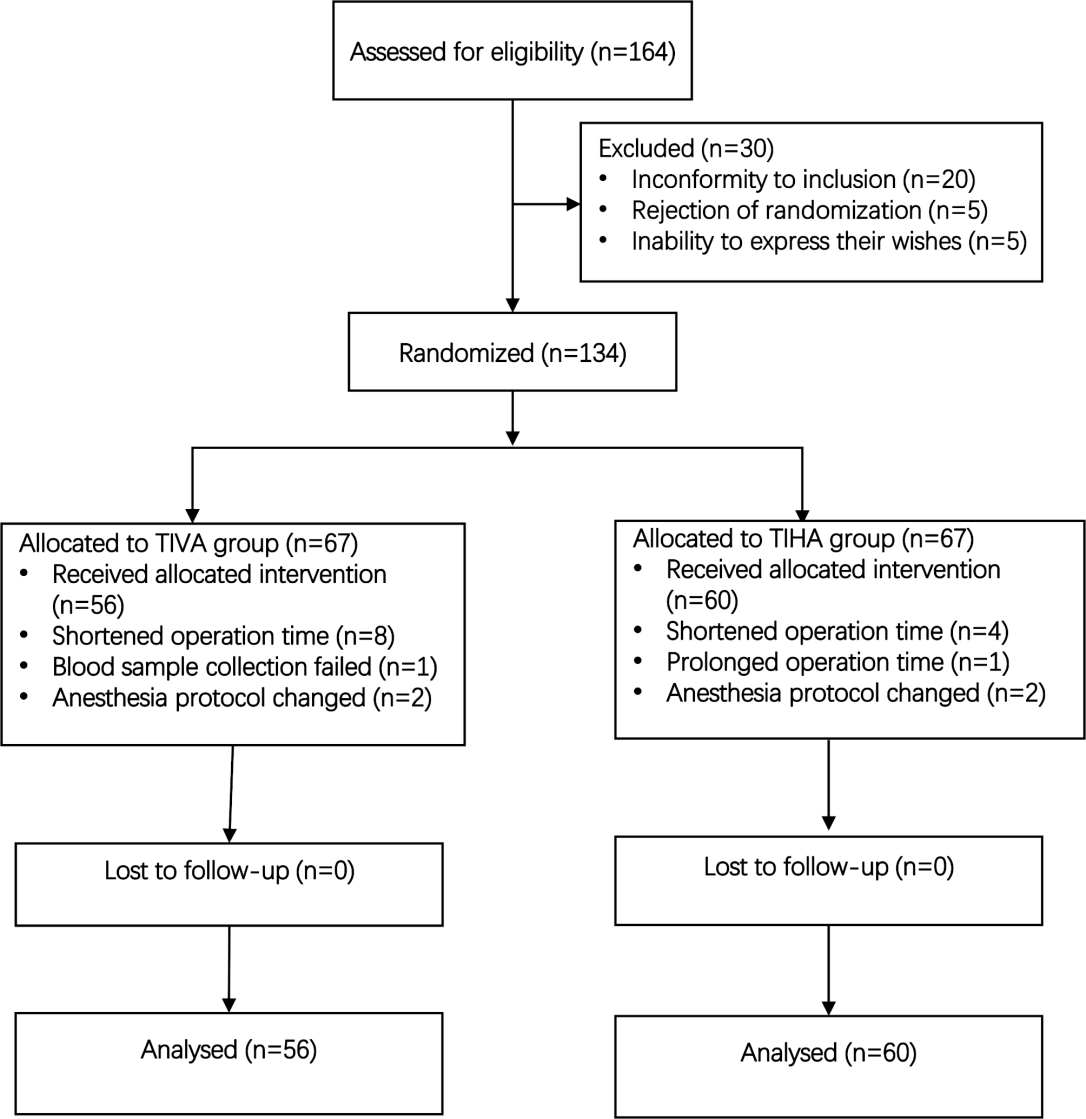
Flow CONSORT diagram of study recruitment TIHA: total intravenous anesthesia, TIHA: total inhalation anesthesia

There were164 patients from January 2020 to October 2020 were screened and 134 participants were enrolled in our trial. A Consolidated Standards of Reporting flow diagram of study recruitment was shown in Fig. 1.

### Patient characteristics

The baseline characteristics of the patients are summarized in Table 1. The average age, sex, BMI value and the preoperative percentage of HbA_1c_ were comparable between the two groups. The perioperative variables are shown in Table 2. There was no significant difference in terms of operation time between the two groups (TIVA 3 [2.2–3.7] vs. TIHA 3 [2.3–3.8]; *P* = 0.158). Notably, the incidence of intraoperative hyperglycemia in TIHA group was higher than that in TIVA group (26.6% [16/60] vs. 10.7% [6/56]; *P* = 0.032).

**Table 1:** Basic Characteristics

	TIVA Group	TIHA Group	*P* Value
	(n = 56)	(n = 60)	
Age (yr)	64 [57, 71]	66 [56, 73]	0.543
Sex (M/F)	33/23	38/22	0.672
Body mass index (kg/m^2^)	23.1 [20.8, 25.5]	23.6 [21.8, 25.6]	0.226
ASA grade (I/II/III)	0/28/28	0/29/31	0.483
Preoperative comorbidities			
Hypertension	31 (55.3)	27 (45.0)	0.265
Coronary artery disease	1 (1.78)	3 (5.0)	0.343
COPD	0	2 (3.3)	0.168
Parkinson’s disease	0	1 (1.6)	0.332
Fasting blood glucose	6.9 [5.3, 8.5]	7.1 [5.1, 9.1]	0.608
Postprandial blood glucose	10.0 [7.9, 12.1]	9.4 [7.5, 11.3]	0.128
Preoperative blood glucose control			
Diet control	7 (12.5)	8 (13.3)	0.894
Insulin	12 (21.4)	9 (15.0)	0.369
Medicine	35 (62.5)	38 (63.3)	0.926
Insulin + medicine	2 (3.6)	5 (8.3)	0.282
Preoperative HBA1c (%)	6.9 [6.5, 8.6]	7.5 [6.6, 8.5]	0.112

The data are expressed as n (%) or median [range]. TIVA, total intravenous anesthesia; TIHA, total inhalation anesthesia; ASA, American Society of Anesthesiologists; COPD, chronic obstructive pulmonary disease; HBA1c, hemoglobinA1c

**Table 2:** Perioperative Variables

	TIVA Group	TIHA Group	*P* Value
	(n = 56)	(n = 60)	
Operation time (h)	3 [2.2, 3.8]	3 [2.3, 3.8]	0.158
Anesthesia time (h)	4.65 [2.8, 6.6]	4.41 [2.6, 6.2]	0.412
Surgery			
Gastrointestinal surgery	27	31	0.710
Liver surgery	16	18	0.866
Biliary tract surgery	4	4	0.919
Thyroid mammary surgery	6	4	0.438
Urinary surgery	3	3	0.931
Intraoperative infusion (ml)			
Crystalloids	1500 [1100, 1800]	1650 [1100, 2075]	0.069
Colloids	500 [500, 1000]	500 [500, 1000]	0.185
Urine output (ml)	500 [200, 700]	450 [300, 738]	0.986
Blood transfusion (n)	4 (7.1)	8 (14.2)	0.222
Remifentanil (µg)	1782 [1130, 2022]	1619 [1008, 1909]	0.903
Sufentanil (µg)	32.5 [27.5, 37.5]	32.5 [27.3, 40.0]	0.535
Insulin use (n)			
Introperative period	5 (8.9)	8 (13.3)	0.452
POD 0	1 (1.8)	6 (10.0)	0.063
POD 1	5 (8.9)	12 (20.0)	0.092
POD 2	3 (5.3)	4 (6.7)	0.767
Hyperglycemia (n)			
Introperation	6 (10.7)	16 (26.6)	0.032
Postoperation	10 (17.8)	20 (33.3)	0.057

The data are expressed as n (%) or median [range]. The *P* values were calculated by the Mann-Whiney U test, chi-square test, or Fisher exact test. TIVA, total intravenous anesthesia; TIHA, total inhalation anesthesia; POD, postoperative day

### Blood glucose level

**Fig. 2:**
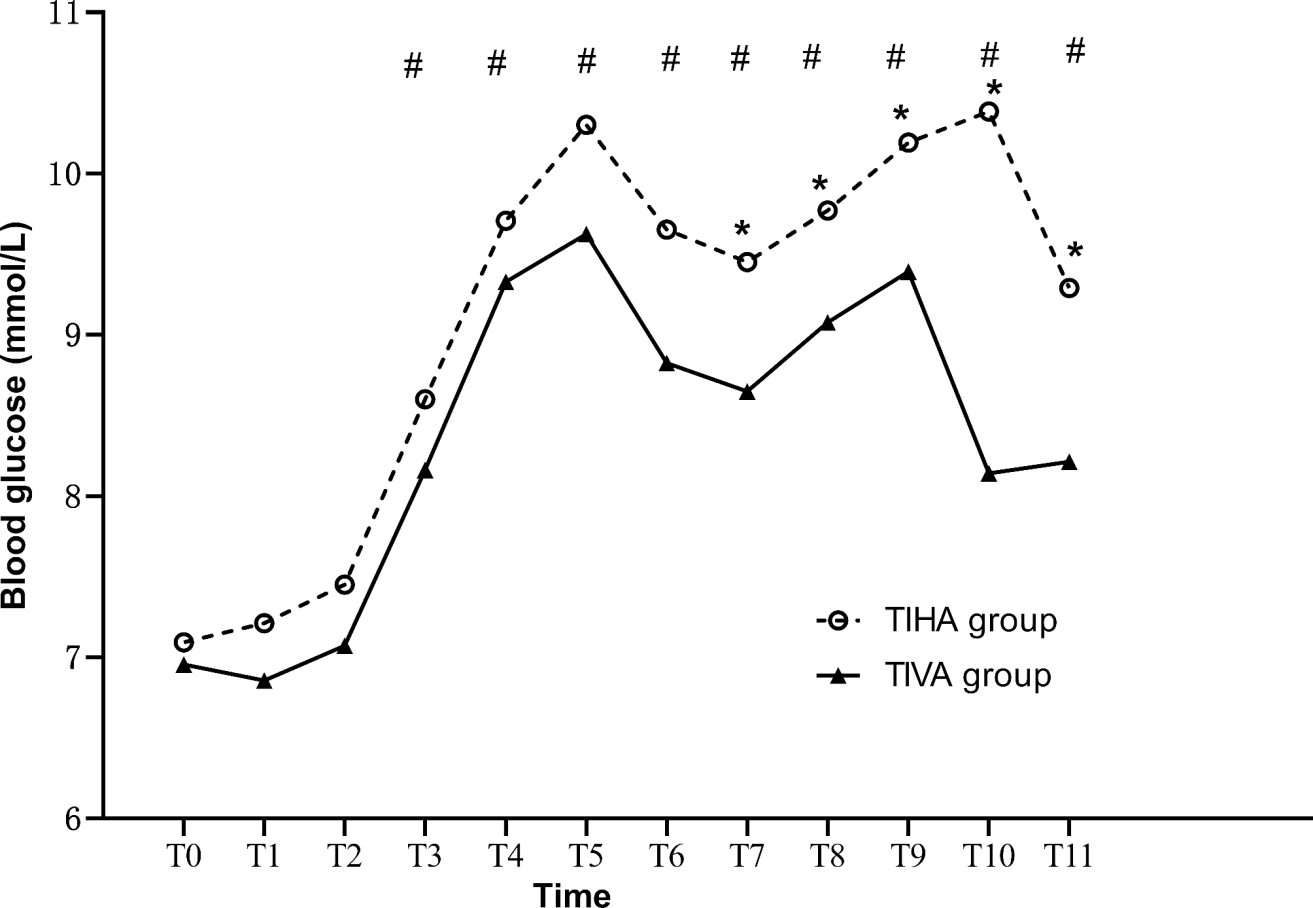
Blood glucose level at different time point in the two groups TIHA: total intravenous anesthesia, TIHA: total inhalation anesthesia, ^*^P < 0.05, compared between the two group; ^#^*P* < 0.05, compared to the baseline level. Time points: T0, preoperative; T1, immediate intubation; T2, skin incision; T3 to T5, 1, 2 and 3 h after the start of the operation, respectively; T6, suturing; T7, extubation; T8, 1 h after the operation; T9, 2 h after the operation; T10, 1 day after the operation; T11, 2 days after the operation

The results showed no interaction between time point and group allocation (*P* = 0.138). Thus, further data analysis was conducted. The blood glucose level of the patients in the two groups were well balanced before surgery. With the prolongation of operation duration, the blood glucose level in both groups gradually increased compared to the baseline level (Fig. 2). However, no statistically significant difference was found between the two groups regarding the blood glucose level at the time points from T0 to T6. The mean [SD] blood glucose level in TIHA group was higher than that in TIVA group at T7- T11, and the difference was significantly at T10 (10.4 ± 2.8 vs. 8.1 ± 2.1 mmol/L ; *P* < 0.01).

### Insulin and cortisol level

**Fig. 3:**
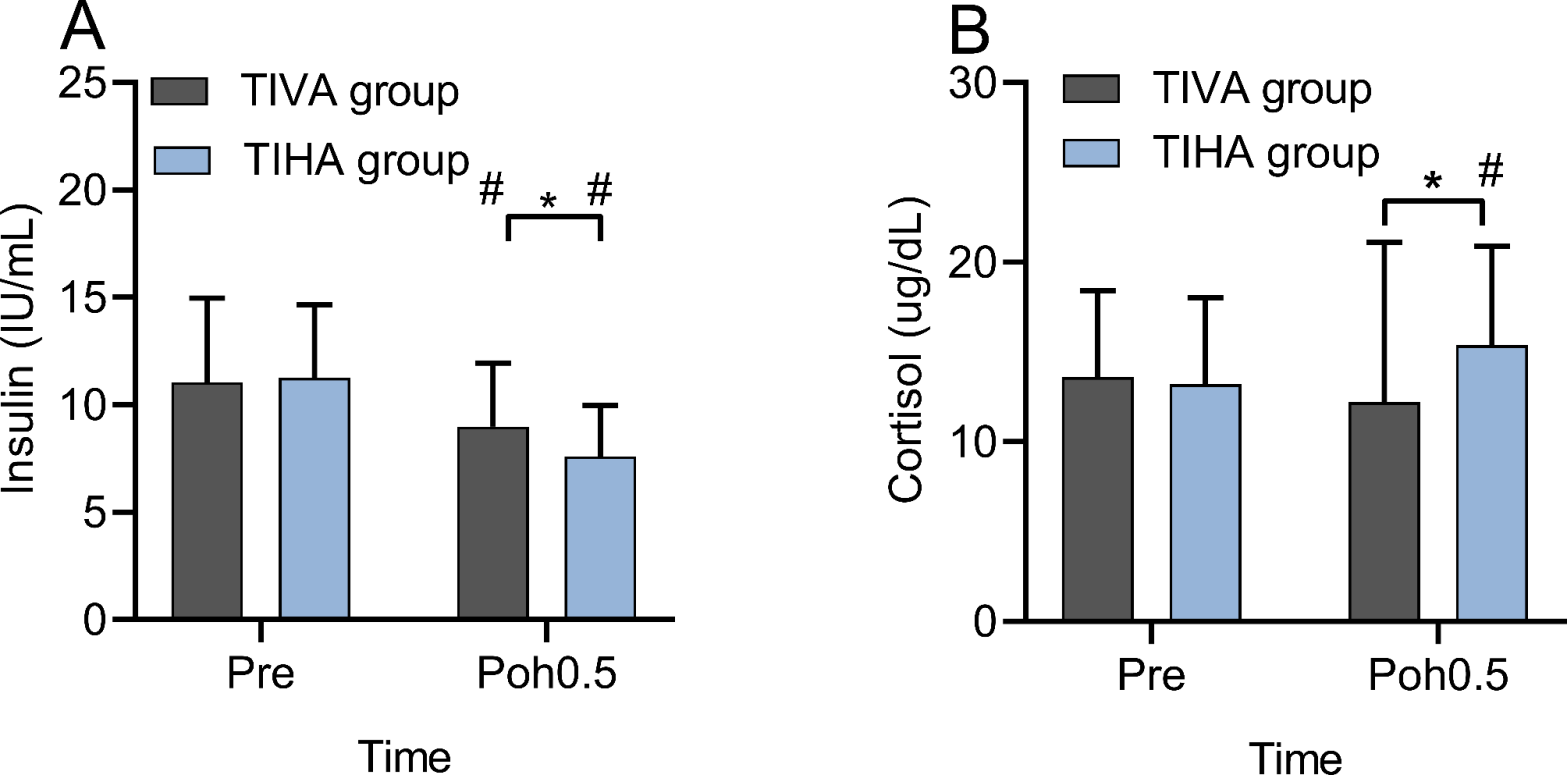
Insulin and cortisol levels of the patients in the two groups TIHA: total intravenous anesthesia, TIHA: total inhalation anesthesia, ^*^P < 0.05, comparison between the two groups; ^#^*P* < 0.05, compared to the baseline level. Pre: preoperative; Poh0.5: 30 min after the operation. A, insulin level; B, cortisol level

Preoperative insulin level in both groups were comparable (11.1 ± 3.9 vs. 11.3 ± 3.4 IU/mL; *P* = 0.652, Fig. 3A). However, the postoperative insulin level in the TIVA group were higher than that in the TIHA group (8.9 ± 2.9 vs. 7.6 ± 2.4 IU/mL ; *P* = 0.011, Fig. 3B). Preoperative cortisol level in both groups were well balanced (13.6 ± 4.8 vs. 13.2 ± 4.8 ug/dL; *P* = 0.701, Fig. 3B). While the postoperative cortisol level in the TIHA group was higher than that in the TIVA group (15.3 ± 4.8 vs.12.2 ± 8.9 ug/dL; *P* = 0.031, Fig. 3B).

### Complications and risk factors

With regards to the postoperative complications, no significant difference was found between the two groups in terms of incidence number of the postoperative complications (TIVA 16% [9/56] vs. TIHA 28% [17/60]; Table 3). A univariate analysis of variables relate to the risk factors for the complications is shown in Table 4. The result demonstrated that risk factors for the complications included group allocation, operation duration, preoperative HBA1c, and the blood glucose level at the time points of 1 h after operation start, 2 h after operation start, 3 h after operation start, suturing, extubation, 1 h after operation, 2 h after operation, and 1 day after operation were associated with postoperative complications. A binary logistic regression analysis was also performed between the abovementioned variables and complications. It showed that only the blood glucose level on the first day after surgery was a risk factor for the postoperative complications (odds ratio [OR], 1.714; 95% confidence interval [CI], 1.008–2.916; *P* = 0.047).

**Table 3:** Incidence of postoperative complications in Patients Who Received Different Types of General Anesthesia

	TIVA Group	TIHA Group	Odds Ratio (95% CI)	*P* Value
	(n = 56)	(n = 60)		
Cardiac insufficiency	0 (0)	1 (1.7)	0.983 (0.951–1.016)	> 0.999
Arrhythmias	1 (1.8)	1 (1.7)	0.932 (0.057–15.269)	> 0.999
PPCS	1 (1.8)	4 (6.7)	3.929 (0.426–36.268)	0.366
Incision infection	0 (0)	2 (3.3)	0.967 (0.992–1.013)	0.496
Anastomotic fistula	1 (1.8)	1 (1.7)	0.932 (0.057–15.269)	> 0.999
Stress ulcer	2 (3.6)	3 (5)	1.421 (0.229–8.837)	> 0.999
Acute kidney injury	1 (1.8)	1 (1.7)	0.932 (0.057–15.269)	> 0.999
Hyperglycemia	3 (5.4)	4 (6.7)	1.262 (0.270–5.906)	> 0.999
Hypoglycemia	1 (1.8)	1 (1.7)	0.932 (0.057–15.269)	> 0.999
Total	9 (16)	17 (28)	2.065 (0.833–5.117)	0.114

The data are expressed as n (%). The P values were calculated by the Fisher exact test or chi-square test. TIVA, total intravenous anesthesia; TIHA, total inhalation anesthesia; PPCS, Postoperative pulmonary complications; There were no significant differences in overall complications in patients received Different Types of General Anesthesia

**Table 4:** Binary logistic regression analysis for the postoperative complications (30d)

Variables	Univariate analysis		Multivariate analysis	
	Odds Ratio (95% CI)	*P* Value	Odds Ratio (95% CI)	*P* Value
Group^a^	2.767 (1.048–7.306)	0.040	2.214 (0.236–5.964)	0.486
Operation time	1.633 (1.086–2.454)	0.018	0.735 (0.140–3.850)	0.716
HBA1c	1.435 (1.050–1.961)	0.023	1.541 (0.596–3.982)	0.372
BMI	0.975 (0.853–1.115)	0.709	−	−
Operation start 1 h glucose	1.266 (1.024–1.566)	0.030	1.442 (0.491–4.234)	0.505
Operation start 2 h glucose	1.238 (1.046–1.465)	0.013	0.852 (0.315–2.305)	0.753
Operation start 3 h glucose	1.330 (1.034–1.710)	0.026	0.968 (0.137–3.559)	0.942
Suturing glucose	1.573 (1.250–1.981)	0.000	0.698 (0.137–3.559)	0.666
Extubation glucose	1.740 (1.333–2.272)	0.000	1.736 (0.353–8.538)	0.497
POH1 glucose	1.551 (1.205–1.997)	0.001	0.278 (0.023–3.306)	0.311
POH2 glucose	1.785 (1.324–2.406)	0.000	5.732 (0.182–6.148)	0.167
POD1 glucose	1.391 (1.162–1.667)	0.000	1.779 (1.009–3.138)	0.047
POD2 glucose	1.227 (0.994–1.515)	0.057	−	−

Group^a^ was dummy variable, TIHA Group=”1”, TIVA=”2”, n = 116. HBA1c, Blood glycated hemoglobin; BMI, body mass index; POH, postoperative hour; POD, postoperative day; −: no analysis performed. Factors of *P* < 0.05 in the univariate analysis were included in the multivariate analysis

## Discussion

In our study, patients with type 2 diabetes in propofol group had lower blood glucose level, less-affected insulin resistance, and better inhibition of cortisol release compared to those in desflurane group. However, no significant difference regarding the incidence of complications between the two groups was found based on the current samples. The pathogenesis of diabetes mellitus is a complicated process, and other systems of the body such as circulatory system, respiratory system and nervous system may be affected at the later stage [29]. However, surgical stress leads to metabolic and neuroendocrine changes, which may cause poor blood glucose control or large blood glucose fluctuation perioperatively, results in adverse outcomes in diabetic patients [10, 30]. Previous studies have shown a correlation between perioperative hyperglycemia and the increase of morbidity and mortality in hospitalized patients [10–12, 31]. Several studies have found that non-diabetic patients anesthetized with isoflurane or sevoflurane have higher perioperative blood glucose levels than those anesthetized with propofol [20, 22, 23, 32].

Majority of earlier studies focused on the effects of propofol and volatile anesthetics on perioperative blood glucose in non-diabetic patients have concluded that the effect of propofol on intraoperative glucose metabolism was less than that of isoflurane and sevoflurane [33, 34]. However, these studies were conducted in non-diabetic patient population regardless of their diabetic status, therefore the result is not accurate in patients with type 2 diabetes. There are several differences between our report and the previous related studies. Our study is a randomized, double-blind, parallel controlled study to determine the impact of total intravenous anesthesia and total inhalation anesthesia as the main maintenance general anesthesia approaches on the blood glucose, cortisol, insulin and postoperative complications in patients with type 2 diabetes, in whom few adequate randomized controlled trials on perioperative blood glucose with type 2 diabetes undergoing abdominal surgery has been conducted. A further difference is that the type of surgery in our study was abdominal surgery, including liver surgery, gastrointestinal surgery and biliary surgery, while previous studies had craniotomy and lung surgery included [20, 23]. In addition, some medications that may affect perioperative blood glucose, such as sugar-containing liquids, glucocorticoids, and non-steroidal analgesics, were not allowed in this study, while in the previous studies, the use of those medications were unknown.

As an influenced factor of surgical stress response, blood glucose indirectly reflects the intensity of stress response [35]. Perioperative surgical stress response may further aggravate metabolic disorders in type 2 diabetes patients. Our study found a rising trend of blood glucose level during the surgery in both TIVA and TIVA groups. Nonetheless, the blood glucose levels in TIVA (propofol) group were lower than that in TIHA (desflurane) group at T7-T11. Previous studies suggested that glucose tolerance and insulin resistance are more prone to the isoflurane and sevoflurane than propofol in type 2 diabetic patients, which resulted in the increase of perioperative hyperglycemia ultimately [33, 34]. Further investigation is required to fully understand the underlying mechanisms of desflurane’s effect on blood glucose.

Cortisol is one of the stress hormones that reflects the intensity of the body’s stress response. Cortisol secretion sensitively varies based on the severity and duration of the surgical trauma throughout the body [22]. The longer the stress duration and the greater the intensity, the higher the serum cortisol level. Cortisol level indicated the degree of body’s stress response [36, 37]. Our study showed that with the prolongation of anesthesia and operation duration, the serum cortisol level increased gradually in both groups, and the postoperative cortisol level in TIHA group was significantly higher than that in TIVA group. One possible explanation could be that propofol inhibited the expression of c-fos gene in central nervous system, thereby inhibiting the activity of the hypothalamic-pituitary-adrenal cortex axis [22].

Previous studies suggested that patients with propofol maintenance anesthesia had a longer postoperative survival time than those with desflurane maintenance anesthesia [38, 39]. ​The underlying mechanism may be the anti-inflammatory and antioxidant properties of propofol, leading to a less likely recurrence of cancer with propofol compared to volatile anesthesia. In our study, no significant difference was found between the two groups in terms of the incidence of postoperative complications. Due to the relative-short term of follow up, it is impossible to compare postoperative survival time in our study. Our study showed that the blood glucose concentration on day 1 after surgery was a risk factor for complications in type 2 diabetics. Notably, the blood glucose concentration of the patients in desflurane group were significantly higher than those in propofol group on the day 1 after surgery. Our results suggest that type 2 diabetes patients with higher blood glucose at day 1 after surgery are more likely to have worse complication. The mechanisms underlying the results could be that poor perioperative blood glucose control may directly affect the function of the patient’s various important organs, which directly affect the patient’s final clinical outcome. Desflurane is commonly used in our hospital which has been reported associate with an increase in the sympathetic tone, and excess concentration was avoided in our study.

A limitation of our study that need to be mentioned is the sample size. The sample size in our study was calculated for blood glucose level comparison, which may not be large enough to show the difference of complications, especially long-term postoperative complications between the two groups. A larger sized trial may be needed to find the difference of postoperative complications between the two group in type 2 diabetic patients.

## Conclusions

TIVA has less impact on perioperative blood glucose level and a better inhibition of cortisol release in type 2 diabetic patients undergoing general surgery compared to TIHA. Due to the relative-short term of follow up, it is impossible to compare postoperative survival time in our study. A future large trial may be conducted to find the difference of long-term complications in type 2 diabetic patients between TIVA and TIHA.

## Data Availability

The datasets used and/or analyzed during the current study are available from the corresponding author on reasonable request.

## Abbreviations

TIVA: Total intravenous anesthesia
TIHA: Total inhalation anesthesia
OR: Odds ratio
CI: Confidence interval
HbA_1c_: HemoglobinA_1c_
EEG: Electroencephalogram
BIS: Bispectral index
S_P_O_2_: Pulse oxygen saturation
MI: Myocardial infarction
PEEP: Positive end expiratory pressure
FiO_2_: Fraction of inspiration O2
PPCS: Postoperative pulmonary complications
EPCO: European perioperative clinical outcome
ANOVA: Analysis of variance
ASA: American Society of Anesthesiologists
COPD: Chronic obstructive pulmonary disease
BMI: Body mass index
POH: Postoperative hour
POD: Postoperative day
