# Correction: Impact of total intravenous anesthesia and total inhalation anesthesia as the anesthesia maintenance approaches on blood glucose level and postoperative complications in patients with type 2 diabetes mellitus: a double-blind, randomized controlled trial

**DOI:** 10.1186/s12871-024-02419-7

**Published:** 2024-01-22

**Authors:** Xinghui Xiong, Yong He, Cheng Zhou, Qin Zheng, Chan Chen, Peng Liang

**Affiliations:** 1 Department of Anesthesiology, West China Hospital, Sichuan University, Chengdu, 610041 Sichuan China; 2 Department of Laboratory Medicine, West China Hospital, Sichuan University, Chengdu, 610041 Sichuan China; 3 Laboratory of Anesthesia and Critical Care Medicine, West China Hospital, National-Local Joint Engineering Research Centre of Translational Medicine of Anesthesiology, Sichuan University, Chengdu, 610041 Sichuan China; 4 Day Surgery Center, West China Hospital, Sichuan University, Chengdu, 610041 Sichuan China

**Correction: BMC Anesthesiol 23, 267 (2023)**

10.1186/s12871-023-02199-6

Following publication of the original article [1], the authors reported an error found under “Intervention and measurement” section. The dose of propofol in continuous infusion used in the TIVA group was 4–12 mg·kg^–1^·h^–1^ instead of 4–12 mg·kg^–1^·min^–1^.

The original article [1] has been updated.
